# Therapeutic effect of Low intensity Extracorporeal Shock Wave Therapy (Li-ESWT) on diabetic bladder dysfunction in a rat model

**DOI:** 10.7150/ijms.55274

**Published:** 2021-01-29

**Authors:** Yung-Chin Lee, Tusty-Jiuan Hsieh, Fang-Hsiang Tang, Jhen-Hao Jhan, Kun-Ling Lin, Yung-Shun Juan, Hsun-Shuan Wang, Cheng-Yu Long

**Affiliations:** 1 Department of Urology, College of Medicine, Kaohsiung Medical University, Kaohsiung, Taiwan; 2 Department of Urology, Kaohsiung Municipal Siaogang Hospital, Kaohsiung Medical University, Kaohsiung, Taiwan; 3 Department of Urology, Kaohsiung Medical University Hospital, Kaohsiung Medical University, Kaohsiung, Taiwan; 4 Regenerative Medicine and Cell Therapy Research Center, Kaohsiung Medical University, Kaohsiung, Taiwan; 5 Graduate Institute of Medicine, College of Medicine, Kaohsiung Medical University, Kaohsiung, Taiwan; 6 Department of Obstetrics and Gynecology, Kaohsiung Municipal Ta-Tung Hospital, Kaohsiung Medical University, Kaohsiung, Taiwan; 7 Graduate Institute of Clinical Medicine, College of Medicine, Kaohsiung Medical University, Kaohsiung, Taiwan; 8 Department of Obstetrics and Gynecology, Kaohsiung Medical University Hospital, Kaohsiung Medical University, Kaohsiung, Taiwan; 9 Department of Urology, Kaohsiung Municipal Ta-Tung Hospital, Kaohsiung Medical University, Kaohsiung, Taiwan; 10 Department of Obstetrics and Gynecology, Kaohsiung Municipal Siaogang Hospital, Kaohsiung Medical University, Kaohsiung, Taiwan

**Keywords:** diabetes mellitus, bladder dysfunction, low intensity extracorporeal shock wave

## Abstract

**Objectives:** Low intensity extracorporeal shock wave therapy (Li-ESWT) has proven to be effective and safe for the treatment of various urological disorders including erectile dysfunction and chronic pelvic pain syndrome. In this study, we elucidated the therapeutic effect and possible mechanisms of Li-ESWT on diabetic bladder dysfunction (DBD) in a rat model.

**Materials and Methods:** In all, thirty-two female Sprague-Dawley rats were divided into three groups: normal control (NC), diabetes mellitus (DM) control, and DM Li-ESWT. The two DM groups were given high fat diets for one month, followed by 2 intraperitoneal injections of streptozotocin (STZ) 30 mg/kg separated by one week. Body weight and fasting blood glucose were monitored every week. Only rats with fasting blood glucose 140 mg/dL or more were considered diabetic and used in the subsequent portions of the study. The Li-ESWTs were applied toward the pelvis of the rats twice a week for 4 weeks with energy flux density (EFD) 0.02 mJ/mm^2^, 500 shocks, at 3Hz. All rats underwent plasma insulin tolerance test, conscious cystometry, leak-point pressure (LPP) assessment, and immunohistochemical studies.

**Results:** DM groups had significantly lower insulin sensitivity and higher body weight. Conscious cystometry also revealed voiding dysfunctions. In the DM Li-ESWT group, the rats had significantly improved voiding functions that were reflected in longer micturition intervals and higher LPP compared to DM control. Immunofluorescence in DM control groups showed increased tyrosine hydroxylase (TH) expression and decreased neuronal nitric oxide synthase (nNOS) expression in the longitudinal urethral smooth muscles. Besides, rats had dilations and deformities of suburothelium capillary network of the bladder, revealing the deterioration of the nerve function of the urethra and destruction of the vascularization of the bladder. However, the DM Li-ESWT group exhibited recovery of the nerve expression of the urethra and vascularization of bladder.

**Conclusions:** Li-ESWT ameliorates the bladder dysfunction and urinary continence in the DBD rat model, reflected in restoration of the nerve expression of the urethra and the vascularization of the bladder. Non-invasive Li-ESWT could be an alternative therapeutic option for DBD.

## Introduction

Diabetes mellitus (DM) is a major healthcare problem affecting 382 million people globally in 2013, with this figure expected to rise to 592 million in 2035 by the International Diabetes Federation 1. DM induces multiple-organ damage and serious sequelae, and causes physical suffering and heavy economic burden. DM is also associated with a broad constellation of urological consequences, including erectile dysfunction (ED) and bladder dysfunction. Over 50% of patients with DM experience diabetic bladder dysfunction (DBD), which can express either through an overactive bladder or underactive bladder syndrome, varies with the severity and duration of diabetes, is described as urgent and frequent, manifesting in incontinence or increased post-void residual urine 2.

The protean nature of DBD is associated with the numerous physiological damages that may occur in the diabetic state, including alteration in smooth muscle cell activity, neuro-vasculopathy, and urothelial dysfunction 3. There are many therapeutic options for voiding dysfunction of DBD, including life style modification, exercise, medicines, rehabilitation and even catherization; however, the treatments of DBD are often refractory due to low therapeutic response or high recurrence rates. Moreover, most of the therapies are focused on the mitigation of the symptoms rather than treatments of the underlying pathologies 4. Thus, alternative treatments, especially those that can restore natural bladder function, are urgently needed.

The deficit and dysfunction of insulin-secreting β-cells are signature symptoms of DM. Dysfunctional β-cells could arise from targeted autoimmune attack (type 1 DM) or multifaceted metabolic disorder associated with sedentary lifestyle and obesity (type II DM). In the past decade, animal models have been extensively used to study the intricacies of pancreas development 5, β-cell physiology and DM 6. More recently, advances in stem cell (SC) technology have provided exciting developments in DM treatment in animal models 7, 8. In terms of DBD, previous studies have shown that adipose tissue-derived stem cells (ADSCs) ameliorate voiding dysfunction in a rat model created by a high-fat diet (HFD) and low-dose streptozotocin (STZ) by reducing apoptosis and improving local neuro-vascularity that are both known to improve tissue function 9; however, regardless of stem cell (SC) type, the clinical implementations of SC therapy still face many challenges, including SC efficiency, potentiality, and safety concerns. More research is still required prior to clinical implementation.

Low-intensity extracorporeal shock-wave therapy (Li-ESWT) has long been used to clinically treat musculoskeletal disorders 10. Li-ESWT has been shown to induce tissue angiogenesis, neuro-regeneration, anti-inflammation, and stem cell activation and recruitment as therapeutic mechanisms 11, 12, and has been validated as effective and safe for the treatment of various urological disorders, including ED and chronic pelvic pain syndrome 13, 14. In previous lupus experiments, Li-ESWT revealed the effect on the immune response in the pancreas, which is the organ most responsible for the cause of DM. In another diabetic rat ED model for Li-ESWT, Li-ESWT significantly reverses diabetes-associated deficits in penile muscle, endothelium and nerve contents, and ameliorates erectile function 15.

The purpose of the study is to elucidate the therapeutic effect and mechanisms of Li-ESWT on DBD in a diabetic rat model. It is proposed that DBD be induced in diabetic rats by administering HFD and multiple low doses of STZ, and then treat the condition with Li-ESWT. These experiments will provide new insights into potential therapies to restore natural bladder function thereby leading to new treatments for DBD.

## Materials and Methods

### Experimental design

**Figure 1:**
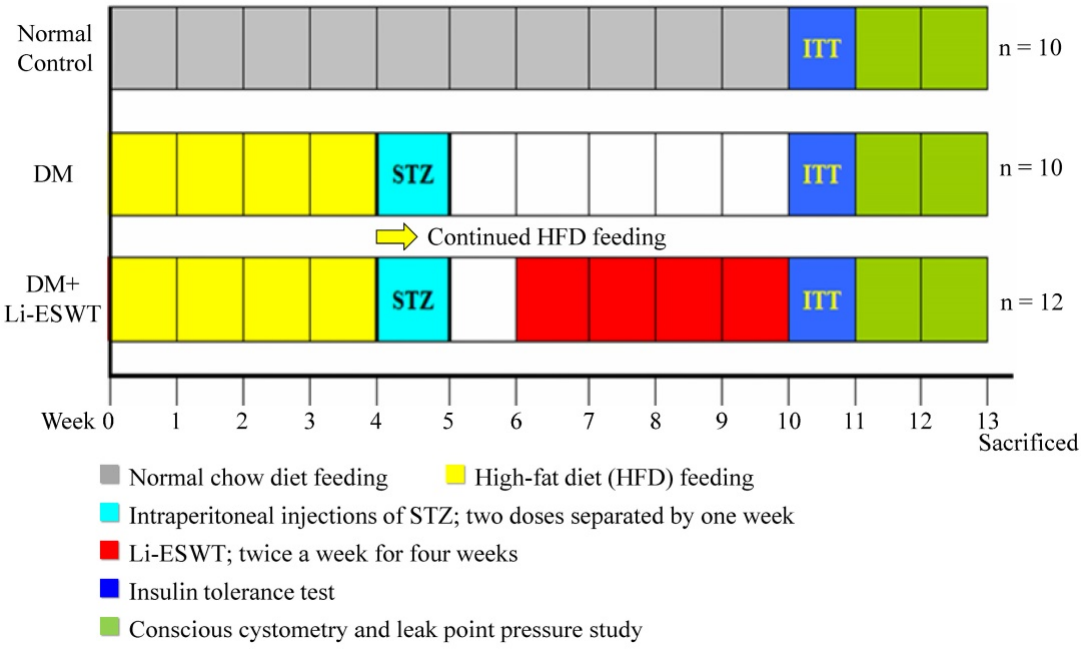
Time flow chart. SW: shock wave. STZ: streptozotocin. ITT: insulin tolerance test.

Thirty-two female Sprague-Dawley rats (8 weeks old) were obtained from Charles River Laboratories (Wilmington, MA, USA). All animal care, treatments, and procedures were approved by the Institutional Animal Care and Use Committee. All rats were given tap water* ad libitum* and maintained in a temperature- and humidity-controlled room on 12-h light/dark cycles, as per the time flow chart of the study shown in Figure 1.

The rats were divided into three groups: normal control group (n=10), DM control group (n=10), and DM Li-ESWT group (n=12). Ten rats fed with standard rat chow served as the normal control group. The remaining 22 rats in diabetic control and Li-ESWT groups were given the high-fat diet (HFD) (ENVIGO) for one month, followed by 2 intraperitoneal injections of STZ (Sigma-Aldrich) at 30 mg/kg separated by one week 16, then allowed to continue feeding on their respective diets until the end of the study. Body weight and fasting blood glucose (rats were fasted for 12h before measurement) were monitored every week. Only rats with fasting blood glucose 140 mg/dL or more were considered diabetic and used in the subsequent portions of the study.

### Low intensity extracorporeal shockwave therapy (Li-ESWT)

The instrumentation was the DUOLITH SD1 T-TOP focused shock wave system (Storz Medical AG). Li-ESWTs were started one week after STZ injection in the DM Li-ESWT group and maintained for four weeks with the energy parameters: energy flux density (EFD) 0.02 mJ/mm^2^, 500 shocks, at 3Hz, twice a week. The rats were prepared under isoflurane anesthesia, placed in the prone position, and the lower abdomen was shaved. Ultrasound gel was applied to engage the Li-ESWT probe with the skin. The LiESWT was applied on the pelvis toward the bladder and urethra. One week after Li-ESWT completion, all the rats received conscious cystometry and leak point pressure studies, and then were sacrificed for tissue harvesting for subsequent histology and immunofluorescent analysis.

### Plasma insulin tolerance test

Five weeks after STZ injection, all rats underwent plasma insulin tolerance test. The rats were fasted for 12 hours with constant access to drinking water. The basal glucose levels were determined in each rat first; next, 0.5 IU/kg of fast-acting insulin (Sigma, St Louis, MO, USA) was administered by intraperitoneal injection. Blood was taken by tail puncture at 30, 60, 90 and 120 min after insulin injection and was measured using test strips (Accu-Check Active; Roche Diagnostics, Indianapolis, IN, USA), with values being presented as a percentage of initial plasma glucose level.

### Conscious cystometry (CMG)

The surgeries for the tube implantations were performed 24 hours before conscious cystometry. Two polyethylene-90 (PE-90) tubes (Clay-Adam, Parsippany, NJ, USA) were prepared in the specific style for intravesical and abdominal pressure measuring. One end of the PE-90 tube was heated to create a collar shape in order to indwell the tube in the bladder; besides, an extra latex balloon was fixed with suture on the same style tube for abdominal pressure measurement. The two tubes were implanted into the bladder and intraperitoneal region separately by placement through a subcutaneous tunnel from the opening on the dorsum of the neck.

On the next day, conscious cystometry proceeded and the rats were placed in the tunnel of the cystometry cage (Braintree Scienticfic, Braintree, MA, USA). The two tubes connected to the pressure transducers (Utah Medical Products, Midvale, UT, USA) attached to the computer with Laboratory view 6.0 software (National Instruments, Austin, TX, USA) to record the pressure continuously; in the meantime, an electric scale was connected to the computer to record the voided volume. Normal saline was infused into the bladder at the rate of 0.1 ml/min using an infusion pump. After stabilizing for 20 minutes, 30 minutes of conscious cystometry were recorded.

The voiding function of each rat was classified as 'abnormal' if bladder filling was accompanied by (i) voiding frequency >12 times/30 min with or without urine leakage (overactive bladder); or (ii) voiding frequency < 6 times/30 min with large bladder capacity (underactive bladder); or (iii) urine leaking continuously without bladder contractions (an acontractile bladder).

### Leak point pressure (LPP) study

Under anesthesia with urethane, the animals were placed at the level of zero pressure and in the supine position, and the lower abdomen was incised to expose the bladder. To determine bladder capacity, the bladder was emptied and then filled with room temperature saline, and at the point of urine leakage, the volume of the infusion was regarded as bladder capacity. The procedure was repeated in triplicate and the average bladder capacity was obtained from these measures.

Leak point pressure was tested while the bladder was infused with half-bladder capacity. Increasing manual extravesical pressure was applied until leakage from the urethra occurred, and the pressure at the moment of urethra leakage was regarded as leak point pressure. This procedure was repeated six times, and the average leak point pressure for each rat was obtained. The rats were subsequently sacrificed, and the organs were harvested for histology.

### Histology and immunofluorescence

The preparation of the bladder and urethra tissue for histology and immunofluorescence were performed according to our previous protocols 17. Primary antibodies, including anti-neuronal nitric oxide synthase (**nNOS**; 1:200; Santa Cruz Biotechnologies), anti-tyrosine hydroxylase (**TH**; 1:200; Millipore Corp, Bedford, MA), and anti-collagen IV (**Col IV**; 1:400; Rabbit, Abcam), were used and counterstained by 4',6-diamidino-2-phenylindole (**DAPI**; Sigma-Aldrich). Secondary antibodies were conjugated with Alexa-488 fluor or Texas Red. Actin was stained by incubation over 20 min with **phalloidin** (Invitrogen) in 4% paraformaldehyde.

### Statistical analysis

Data were analyzed with Prism 5 (GraphPad Software, Inc.) and expressed as mean ± standard error (SE) of the mean for continuous variables. Continuous data were compared among the groups by using one-way analysis of variance while the Tukey-Kramer test was used for post-hoc comparisons. To evaluate the effect of Li-ESWT among the groups, the chi-square test was performed with Fisher's Exact Test. Statistical significance was set at *P* < 0.05.

## Results

### Biological characteristics of the rats

**Figure 2:**
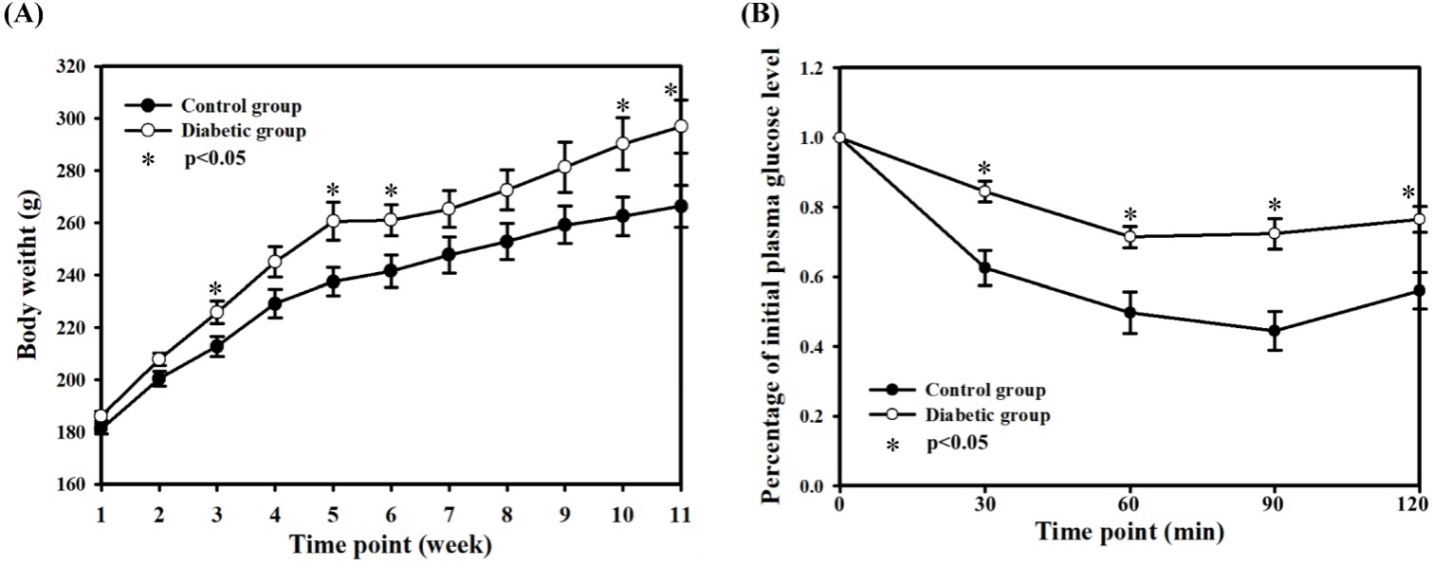
Body weights (A) and insulin tolerance test (B). The body weights were recorded weekly. The insulin tolerance test was performed after 5 weeks of STZ injection. The data are mean ± SD. Normal control group: n = 10; diabetic group: n = 22.

Weekly body weight revealed significantly higher body weight in the two DM groups compared with the normal control group, at a mean (SE) of 266.4 (8.0) g for the normal control group vs 296.9 (10.3) g for the DM groups (*P* < 0.05, at the 11th week) (Fig. 2A). After three weeks of HFD feeding, the rats in the diabetic groups had heavier body weight. In the ITT experiment, animals in the diabetic groups had lower insulin sensitivity relative to the normal control group (Fig. 2B), although there were no statistical differences between the two diabetic groups. The results revealed the consistency and the successful induction of a type II diabetic model in this experiment, suggesting that the current diabetic situation would be similar to the pathophysiological state of type II DM in humans, consistent with previous results.

### Li-ESWT ameliorated bladder dysfunction and improved urinary continence

**Figure 3:**
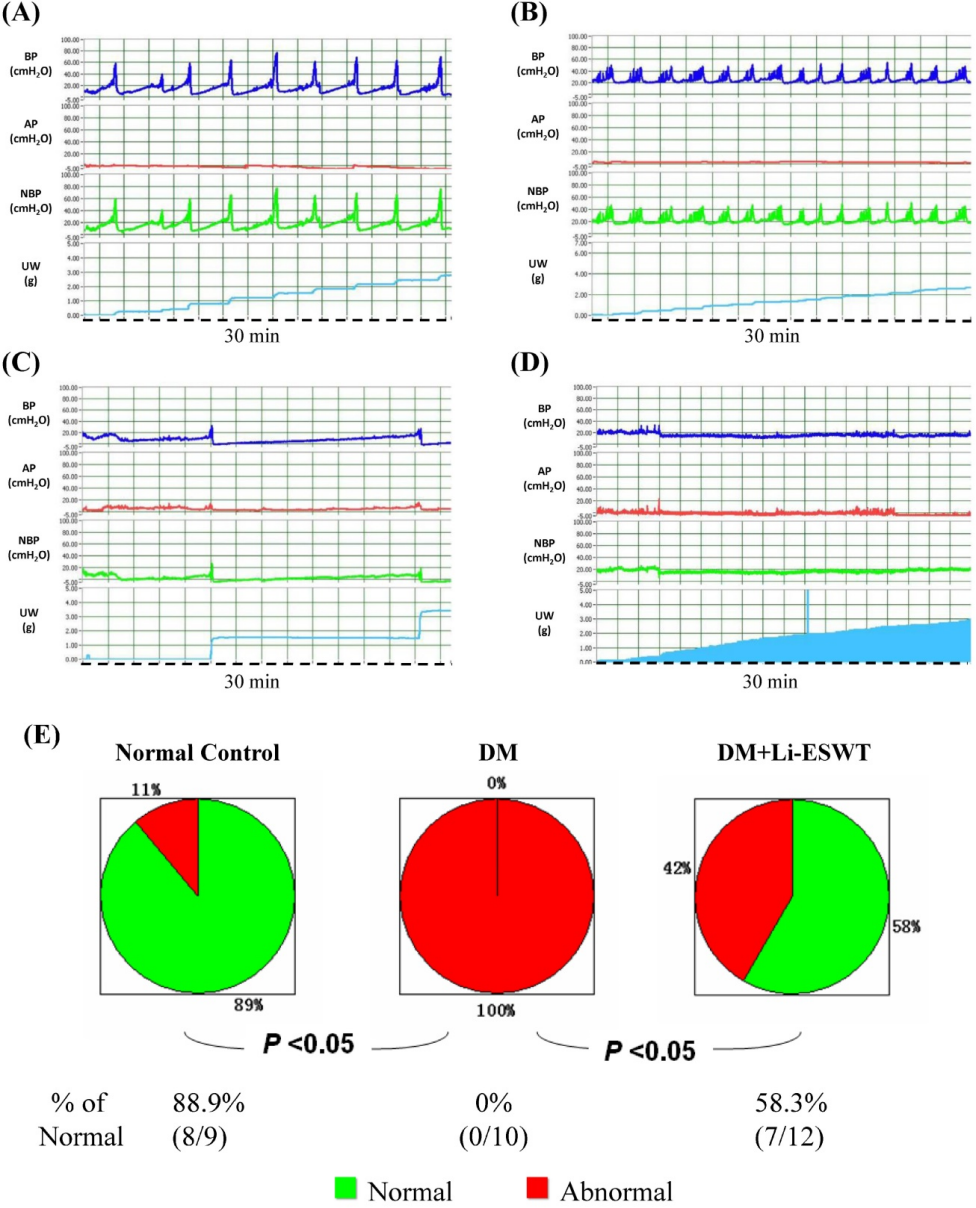
Assessment of voiding function with conscious cystometry. (A) Normal voiding pattern was noted in 9 rats of the control group and 7 rats of the DM+Li-ESWT group. (B) Overactive bladder pattern was noted in one rat of the control group, 6 rats of the DM group and one rat of the DM+Li-ESWT group. (C) Underactive bladder pattern was noted in 2 rats of the DM group and 2 rats of the DM+Li-ESWT group. (D) Acontractile bladder pattern was noted in 2 rats of the DM group and 2 rats of DM+Li-ESWT group, which was also regarded as abnormal voiding pattern. (E) Pie charts of the voiding patterns. Normal voiding patterns were significantly more common (P < 0.05) in the normal control group compared with the DM group. A significant proportion of the DM rats that received Li-ESWT had normal voiding patterns. BP: bladder pressure (cmH2O). AP: abdomen pressure (cmH2O). NBP: net bladder pressure (cmH2O). UW: urine weight (g). DM: diabetes mellitus.

Representative cystometric graphs of normal and abnormal voiding patterns are shown in Figure 3A-D. All the rats in the normal control group manifested a normal voiding pattern, with the frequency of 6 to 12 times per 30 min during cystometry. For each micturition cycle, the bladder pressure gradually increased from 0 to 20 cmH_2_O at the filling phase and highly peaked at 47.9 ± 5.7 cmH_2_O during voiding. A discrete urine output pattern with amount of 0.4 ± 0.1 ml was found for each bladder contraction in the normal control group.

For the DM control group, all the rats revealed abnormal urination, with six rats (n = 6) exhibiting an overactive bladder pattern of voiding frequency being more than 12 times per 30 min, bladder voiding pressure at 49.0 ± 13.9, and discrete voiding amount of 0.27 ± 0.19 ml. Another four rats in the DM control group revealed different types of voiding dysfunction: 2 in underactive bladder and 2 in acontractile bladder (Fig. 3C, D). Underactive bladder was defined as longer micturition intervals and larger micturition volume per void compared with the normal pattern, whereas acontractile bladder pattern was defined as continuous urine leakage without obvious bladder contractions during filling phase.

In the DM Li-ESWT group, a significantly increased percentage of rats with normal voiding pattern were found compared to the DM control group (58.3 % of the DM Li-ESWT group compared with 0 % of the DM control group, *P* < 0.05, Fig. 3E). Seven rats (58.3%) in the DM Li-ESWT group showed normal voiding pattern after Li-ESWT, whereas 1 showed overactive bladder, 2 showed underactive bladder and 2 showed acontractile bladder. Significantly longer micturition intervals and larger LPP in the DM Li-ESWT group compared to DM controls (Table 1) were also found. These cystometry and LPP results suggested that Li-ESWT ameliorated bladder dysfunction and urinary incontinence.

### Li-ESWT restored nerve expression of the urethra

Immunofluorescence with TH and nNOS were performed for the evaluation of the pathophysiological changes of the nerve after Li-ESWT. TH, as a classical marker of presynaptic sympathetic innervation, innervates urethral longitudinal smooth muscle contraction, and operates urination. Overexpression of TH reflects the dysfunction of urethral continence, causing urinary incontinence. In the current experiments, TH expressions of the urethral longitudinal smooth muscles were increased in the DM control group and recovered in the DM Li-ESWT group. On the other hand, nNOS, one of nitric oxide synthases, produces NO in the nerve of the urethra, induces relaxation of the urethral longitudinal smooth muscle, and controls urinary continence. Decreased nNOS expression results in dysfunction of urethral continence and causes urinary incontinence. In this study, nNOS expression was decreased in the DM control group and recovered in the DM Li-ESWT group. The immunofluorescence suggested that Li-ESWT restored nerve expression of the urethra and improved urinary incontinence.

### Li-ESWT activated angiogenesis of the bladder

**Figure 4:**
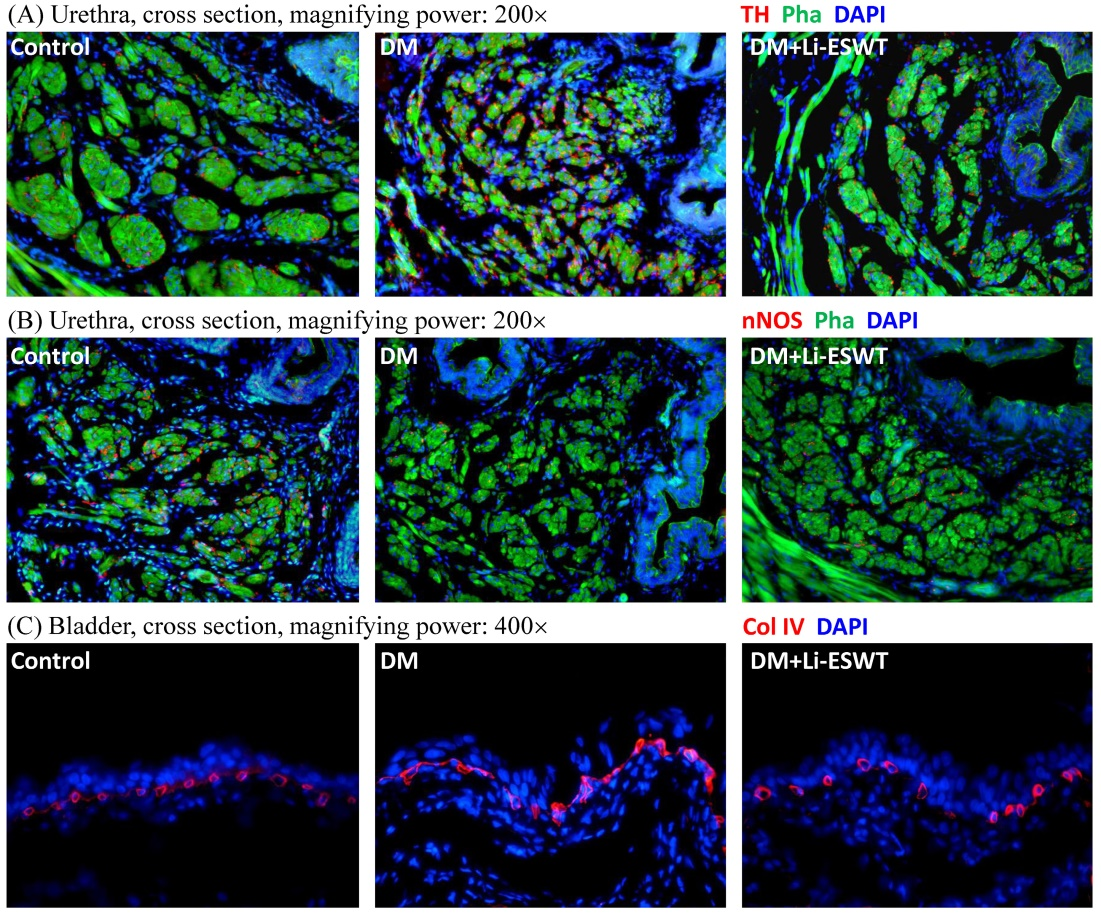
(A) Representative cross sectional images of the urethral smooth muscles stained with Alexa 488 conjugated phalloidin (Pha; green fluorescence) and the sympathetic nerves stained with anti-tyrosine hydroxylase (TH; red fluorescence) antibody. Compared to the control group, urethras in the DM group demonstrated higher TH expression in longitudinal smooth muscles, which was improved after Li-ESWT. (B) Representative cross sectional images of the urethral smooth muscles stained with Alexa 488 conjugated phalloidin (Pha; green fluorescence) and the nitric nerves stained with anti-neuronal nitric oxide synthase (nNOS; red fluorescence) antibody. Compared to the control group, urethras in the DM group showed lower nNOS expression in longitudinal smooth muscles, which was improved after Li-ESWT. (C) Representative images of the bladder suburothelium stained with anti-collagen IV (Col IV; red fluorescence) antibody. Compared to the normal controls, dilations and deformities of suburothelium capillary network were noted in the DM rats, which were improved after Li-ESWT. All the tissue sections were counterstained with 4',6-diamidino-2-phenylindole (DAPI; blue fluorescence) to identify the cell nuclei.

Immunofluorescence with Col IV of the bladder (Fig. 4C) reflected the vascularity in the bladder suburothelium related to Li-ESWT. Compared with the normal control group, rats in the DM group showed dilations and deformities of the suburothelium capillary network, indicating the destruction of bladder vessels due to diabetes. After Li-ESWT, the recovery of the integrity of the blood vessels in the bladder suburothelium was found in the DM Li-ESWT group. The findings suggested that Li-ESWT could activate angiogenesis and recovery of the vascularity of the bladder.

## Discussion

Diabetes is one of the most common metabolic disorders that results in a state of persistent hyperglycemia. About 5-10% of affected people are diagnosed as type I, and approximately 90-95% of subjects are type II. Diabetes may be related to the environment, diet, genetics, autoimmunity, and possibly other as-yet-undetermined causes. Although many animal models for type 2 diabetes have been developed, models using only one method inducing the diabetic state might not reflect the actual conditions of human subjects 16. Modified animal models with combination of HFD and multiple low doses of STZ injection have revealed efficiency in generating rat models that mimic the natural history and metabolic characteristics of type 2 diabetes in humans 16, 18. In this study, we successfully induced a type II DM rat model with HFD and two low doses of injected STZ, and maintained the consistency of DM for each DM group.

DBD is a progressive disease. The symptoms and the progressions of DBD vary with the severity and duration of diabetes. Urinary dysfunction of DBD might take the form of an underactive bladder with sensory deficit and poor contractility, or an overactive bladder with micturition typically described as urgent and frequent, with or without incontinence 2. In the current study, most of the rats in the DM group had cystometric findings consistent with overactive bladders. According to “DBD Time Theory” reported from Daneshgari's study 19, DBD might express OAB symptoms in the early phase, and then subsequently transform to underactive bladder in the late phase. Our diabetic rat model was more consistent with early-stage DBD.

Based on cystometric analysis, it was found that all the rats in the DM group had voiding dysfunction compared to only 41.7% in the DM Li-ESWT group. Nearly 60% of the DM rats exhibited recovery of bladder dysfunction after Li-ESWT. These findings reveal that Li-ESWT ameliorates the voiding dysfunctions caused by DM. The ameliorated rate in our study was similar to previous research in regard to direct bladder injection with adipose tissue-derived stem cells (ADSCs) in a type 2 diabetic rat model 9. In addition, an LPP study revealed decreased LPP in a DM group and recovered LPP after Li-ESWT in a DM Li-ESWT group, indicating that the DM rats expressed urinary incontinence and Li-ESWT recovered the continence function. Another study had consistent findings that Li-ESWT was used for late phase of DBD rats with underactive bladder (UAB) 26.

In DM patients, physiological damage including impaired smooth muscle, neuropathy and urothelial dysfunction result from persistent hyperglycemia, which in turn leads to DBD 3. As above, in our results, the expression of TH (as a classical marker of presynaptic sympathetic innervation) in the longitudinal smooth muscle of the urethral section in the DM group was significantly increased, and the expression of nNOS in DM group was significantly decreased, resulting in the imbalance of muscle coordination of the urinary function in the DM group causing DBD and urinary incontinence. Urinary functions are operated by coordination of multiple corresponding muscles including the bladder detrusor muscle and the urethral longitudinal and circular smooth muscle during storage and voiding phase of the micturition cycle. Hyperglycemia and peroxide toxin induced by DM destroys nerve and muscle function causing imbalance of nerve innervation and function, resulting in DBD. Li-ESWT restores the nerve function of the bladder and urethra, and ameliorates the voiding dysfunction of DBD. Meanwhile, DM rats had dilations and deformities of the suburothelium capillary network in the bladder section, revealing damage of bladder vascularity by DM. Li-ESWT restored the capillary network of the bladder and improved vascularization of the bladder. These findings suggest mechanisms of therapeutic effect of Li-ESWT in the promotion of angiogenesis and re-innervation and recovery of the nerve function.

A variety of Li-ESWT mechanisms have been proposed, including angiogenesis 20, tissue regeneration, nerve re-innervation 15, 21, 22, stem cell activation and recruitment 23, 24, and anti-inflammation 25. In the study of Li-ESWT in UAB by Wang et al. 26, Li-ESWT ameliorates the UAB, reflected in activated nerve innervation and enhanced muscle regeneration and bladder contractile function. Immunofluorescence staining of anti-S100 antibody (labeling of Schwann cells in the peripheral nervous system) showed that DM caused neurological damage and decreased nerve distribution, while Li-ESWT recovered nerve expression. The results showed that Li-ESWT contributes to nerve re-innervation, thereby restoring bladder nerve function. In the Li-ESWT in ED study, Li-ESWT promoted neurite regeneration by increasing nNOS-positive neurons, thereby improving erectile dysfunction 15. The study investigating the mechanism of Li-ESWT in ED showed that the protein kinase RNA-like endoplasmic reticulum kinase (PERK) and the transcription factor 4 (ATF4) pathway could enhance the expression of brain-derived neurotrophic factor (BDNF) and promote nerve regeneration in Schwann cells 22. In addition, Li-ESWT could also activate urethral muscle-derived stem cells, enhance myogenesis, and improve urinary incontinence by activating the PERK/ATF4 pathway 27.

Angiogenesis by Li-ESWT is one of the key mechanisms of Li-ESWT. Vascular endothelial growth factor (VEGF) plays an important role in the angiogenesis of Li-ESWT. In the studies of Li-ESWT in wound repair, Li-ESWT enhances neovascularization by increasing the expression of VEGF and endothelial nitric oxide synthase (eNOS), inhibits inflammatory response by limiting leukocyte infiltration, improves skin flap tissue survival, and promotes wound healing 20, 25.

There are some limitations in the current study. Firstly, although there were promising results of urinary function related to nerve function recovery and vessel angiogenesis, mechanisms regarding muscle and stem cell aspects were not examined in this study. Previous studies have shown promising results of Li-ESWT in promoting muscle regeneration and enhancing muscle contractile function, activation and recruitment of stem cells 23,26. Secondly, under the current dosage of Li-ESWT, this study showed positive results for DBD, as the dosages and the patterns of Li-ESWT correlated with the therapeutic effect and safety. Different Li-ESWT models, focused or defocused, also affect energy conduction and influence the results. Further studies related to dosage and safety issues should be conducted.

## Conclusion

Li-ESWT ameliorated bladder dysfunction and urinary continence in the DBD rat model, reflected in restoration of the nerve expression of the urethra and the vascularization of the bladder. Non-invasive Li-ESWT could be an alternative therapeutic option for DBD.

## Figures and Tables

**Table 1:** Comparison of voiding parameters in conscious cystometry

	Normal control	DM	DM+Li-ESWT
Max voiding pressure (cm-H_2_O)	47.9 ± 5.7	49.0 ± 13.9	45.6 ± 11.8
Micturition interval (s)	385.3 ± 124.6	225.0 ± 112.8*	377.1 ± 230.4**
Urine volume/void (ml)	0.40 ± 0.1	0.27 ± 0.2	0.64 ± 0.4
LPP (cm-H_2_O)	42.8 ± 11.6	28.0 ± 11.4*	38.7 ± 17.2**

Data are mean ± SD; *p<0.05 versus normal control group; **p<0.05 versus DM group.
